# EXAMINING THE CUMULATIVE EFFECTS OF SOCIAL ISOLATION AND LONELINESS IN OLDER ADULTS WITH HIV

**DOI:** 10.1093/geroni/igac059.1433

**Published:** 2022-12-20

**Authors:** Moka Yoo-Jeong

**Affiliations:** Northeastern University, Melrose, Massachusetts, United States

## Abstract

Social isolation exists when one has limited or lacks social contact with others and is distinct from loneliness, an affective state on the perception of isolation. Social isolation and loneliness are recognized as risks to well-being among older adults. Less in known about the cumulative effects of social isolation and loneliness in older persons with HIV (OPWH). Using cross-sectional data on OPWH (age ≥50) recruited from an outpatient HIV clinic in Atlanta, GA (N=146), we aimed to 1) describe the overlap between social isolation and loneliness and 2) examine the combined effects of social isolation and loneliness on quality-of-life (QoL), HIV-related stigma, depressive symptoms, and comorbidity burden. Loneliness and social isolation were assessed using the PROMIS-Social Isolation Scale and Social Network Index, respectively. Participants were grouped into four categories into ‘lonely only,’ ‘isolated only,’ ‘both lonely and isolated,’ or ‘neither.’ Bivariate and adjusted associations were conducted. Among participants (mean age=56.53), 26.7% (n=39) were considered ‘lonely’ only, 12.3% (n=18) ‘isolated’ only, 15.1% (n=22) ‘both lonely and isolated,’ and 45.9% (n=67) ‘neither.’ In bivariate analyses, individuals categorized as ‘both lonely and isolated’ were likely to have past homelessness and higher depressive symptoms, stigma, and comorbidity burden, and lower QoL. In adjusted models, ‘both isolated and lonely’ significantly predicted QoL, stigma, and depressive symptoms. Findings highlight the critical emphasis on targeting OPWH who are both isolated and lonely.

